# Effect of Hepatitis C Virus Genotype 1b Core and NS5A Mutations on Response to Peginterferon Plus Ribavirin Combination Therapy

**DOI:** 10.3390/ijms160921177

**Published:** 2015-09-07

**Authors:** Shingo Nakamoto, Fumio Imazeki, Makoto Arai, Shin Yasui, Masato Nakamura, Yuki Haga, Reina Sasaki, Tatsuo Kanda, Hiroshi Shirasawa, Osamu Yokosuka

**Affiliations:** Department of Gastroenterology and Nephrology, Graduate School of Medicine, Chiba University, Chiba 260-8677, Japan; E-Mails: imazekif@faculty.chiba-u.jp (F.I.); araim-cib@umin.ac.jp (M.A.); ntcph863@yahoo.co.jp (S.Y.); masato.nm@hotmail.co.jp (M.N.); hagayuki@gmail.com (Y.H.); reina_sasaki_0925@yahoo.co.jp (R.S.); kandat@aol.com (T.K.); sirasawa@faculty.chiba-u.jp (H.S.); yokosukao@faculty.chiba-u.jp (O.Y.)

**Keywords:** hepatitis C virus, core region, interferon sensitivity determining region, peginterferon, sustained virologic response

## Abstract

We examined whether hepatitis C virus (HCV) genotype 1b core- and NS5A-region mutations are associated with response to peginterferon α-2b plus ribavirin combination therapy. A total of 103 patients with high HCV genotype 1b viral loads (≥100 KIU/mL) were treated with the combination therapy. Pretreatment mutations in the core region and interferon sensitivity determining region (ISDR) in the NS5A region were analyzed. In univariate analysis, arginine and leucine at positions 70 and 91 in the core region, defined as double wild (DW)-type, were associated with early virologic response (*p* = 0.002), sustained virologic response (SVR) (*p* = 0.004), and non-response (*p* = 0.005). Non-threonine at position 110 was associated with SVR (*p* = 0.004). Multivariate analysis showed the following pretreatment predictors of SVR: hemoglobin level ≥ 14 g/dL (odds ratio (OR) 6.2, *p* = 0.04); platelet count ≥ 14 × 10^4^/mm^3^ (OR 5.2, *p* = 0.04); aspartate aminotransferase (AST)/alanine aminotransferase (ALT) ratio < 0.9 (OR 6.17, *p* = 0.009); DW-type (OR 6.8, *p* = 0.02); non-threonine at position 110 (OR 14.5, *p* = 0.03); and ≥2 mutations in the ISDR (OR 12.3, *p* = 0.02). Patients with non-DW-type, non-threonine at position 110, and <2 ISDR mutations showed significantly lower SVR rates than others (11/45 (24.4%) *vs.* 27/37 (73.0%), respectively; *p* < 0.001). SVR can be predicted through core and NS5A region mutations and host factors like hemoglobin, platelet count, and AST/ALT ratio in HCV genotype 1b-infected patients treated with peginterferon and ribavirin combination therapy.

## 1. Introduction

Hepatitis C virus (HCV) affects an estimated 170 million individuals worldwide and is one of the most frequent causes of chronic hepatitis, cirrhosis, and hepatocellular carcinoma [1].

A combination of peginterferon and ribavirin has long been used for the treatment of chronic HCV infection [2,3]. Recently, direct acting antivirals (DAAs) have been developed for HCV treatment and many regimens are available; however, efficacy of DAAs is genotype dependent and the emergence of drug resistant mutation is a problem for these drugs. It is likely that interferon-based therapy is still an important option for such patients as failed to respond to DAAs-based therapy. A combination of peginterferon α and ribavirin achieved a sustained virologic response (SVR) rate of approximately 50% in patients infected with high HCV genotype 1 viral load, the dominant type in Japan [4], Europe, and US [5,6,7]. Response to interferon-based therapy has been shown to depend on interleukin 28B (*IL28B*) gene polymorphism, HCV genotype, viral load, ethnicity, age, hepatic fibrosis, hepatic steatosis, and drug adherence [8,9,10,11]. The variation of IL28B which encodes for interferon lambda has also been associated with response to interferon-free therapy, indicating the importance of endogeneous interferon on HCV treatment [12].

Besides these predictors, several studies have indicated that mutations in the functional regions of HCV proteins may be correlated with the response of HCV genotype 1b to interferon therapy, such as mutations of amino acids at positions 70 in the HCV core region or interferon sensitivity determining region (ISDR) of the NS5A region in Japan, US, and Europe [13,14,15,16,17]. HCV core has been reported to interact with many cellular factors. Type I interferon (interferon α and interferon β) binds to the type I interferon receptors, which, in turn, activates Janus kinase (Jak)-signal transducer and activators of transcription (STAT) pathways. It was reported that HCV core protein inhibits these pathways [18,19,20]. To date, few studies have examined outside position 70 in the core and the entire core sequence on response to interferon-based therapy.

In this study, we determined the amino acid patterns of the entire core and ISDR of NS5A regions in Japanese patients infected with genotype 1b-HCV and analyzed the association between these mutations and the virological response to peginterferon plus ribavirin combination therapy. We also examined the relationship between viral mutations and host factors.

## 2. Results

### 2.1. Response to Therapy

EVR and NR were achieved by 72 of 99 (72.7%) and 25 of 103 (24.3%) patients, respectively. EVR and NR rates in the extended treatment group (15/19 (79%) and 4/21 (19%), respectively) did not differ from those in the standard treatment group (57/80 (72%) and 21/82 (26%); *p* = 0.50 and 0.53, respectively). Among 82 patients in the standard treatment group, 38 (46.3%) achieved SVR. Of these, 57 of 81 (70.4%) achieved EVR, and the SVR rate in patients with EVR (37/57 (64.9%)) was significantly higher than that in patients with non-EVR (1/24 (4.2%), *p* < 0.0001).

### 2.2. Specific Amino Acid Patterns in the Core Region According to Virological Response

**Figure 1:**
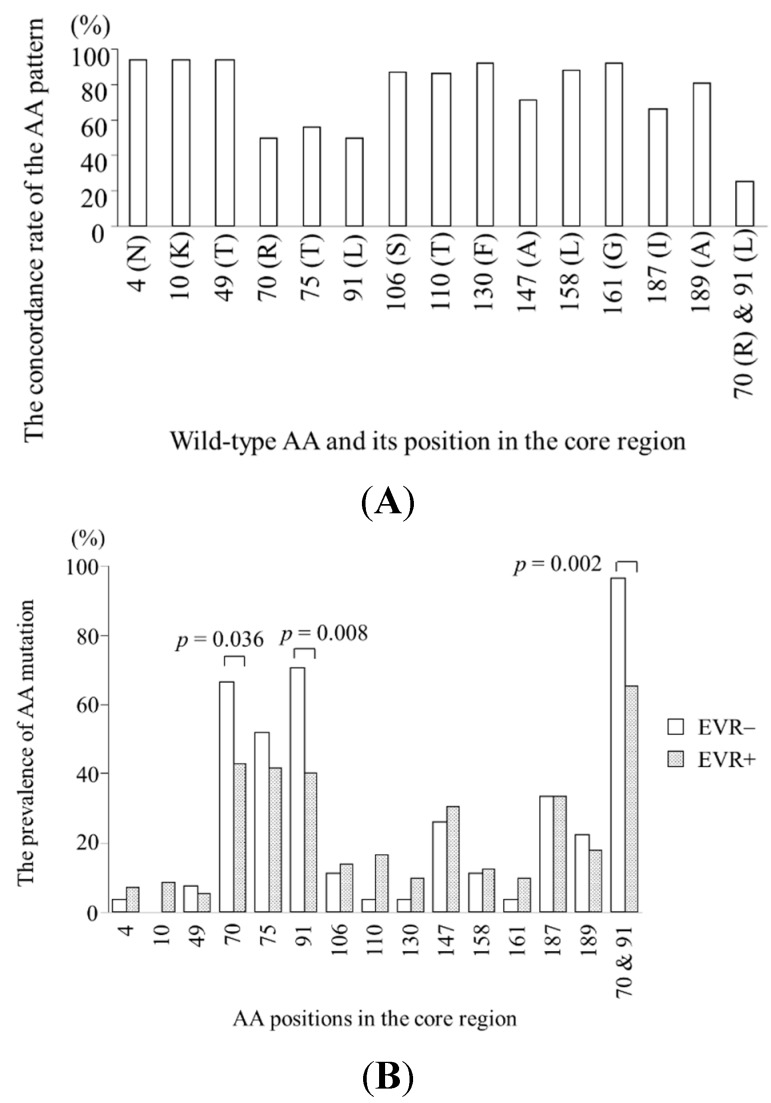

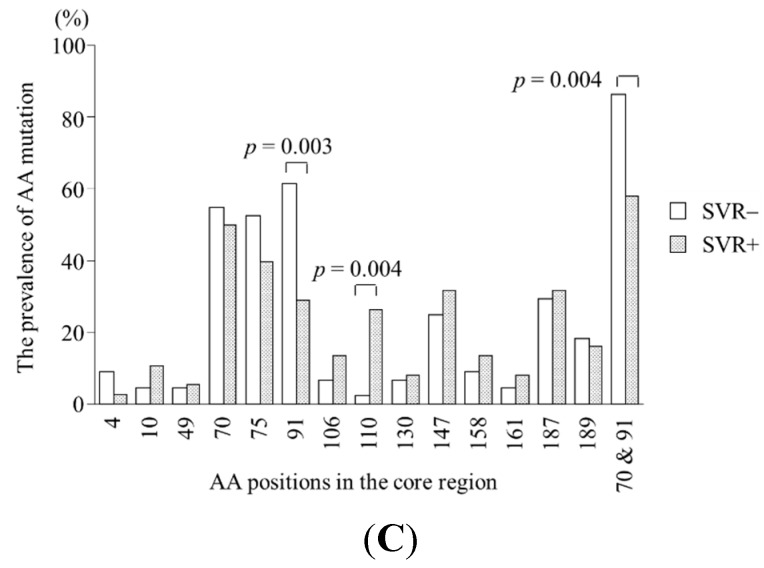
Specific amino acid patterns in the core region of hepatitis C genotype 1b patients. At 14 of 191 positions, the most prevalent (wild-type) amino acid accounted for 50%–95% of the 103 sequences (**A**); The amino acid mutation rates of these positions between early virologic response (EVR) and non-EVR patients (**B**); and those between sustained virologic response (SVR) and non-SVR patients (**C**); *p* value is shown in the positions with significant difference in amino acid pattern between both groups; AA, amino acid.

Each amino acid position (1–191) of core sequences derived from the 103 patients was analyzed separately, revealing that most positions were highly conserved: at 177 of 191 positions, the most prevalent (wild-type) amino acid accounted for >95% of the entire sequences and no association was observed with virological response. At remaining 14 positions, wild-type amino acid accounted for 50%–95% (Figure 1A). Figure 1B,C shows the amino acid mutation prevalence at these 14 positions according to virological response: the mutation rate at position 70 was significantly higher in non-EVR patients than in EVR patients (67% *vs.* 43%, *p* = 0.036). The mutation rate at position 91 was significantly higher in non-EVR patients than in EVR patients (70% *vs.* 40%, *p* = 0.008) and higher in non-SVR patients than in SVR patients (61% *vs.* 29%, *p* = 0.003). The prevalence of non-DW-type amino acids at positions 70 and 91 was significantly higher in non-EVR patients than in EVR patients (96% *vs.* 65%, *p* = 0.002) and in non-SVR patients than in SVR patients (86% *vs.* 58%, *p* = 0.004). The mutation rate at position 110 was significantly higher in SVR patients than in non-SVR patients (26% *vs.* 2%, *p* = 0.004). Therefore, amino acid patterns at positions 70, 91, and 110 were further investigated.

### 2.3. Viral Kinetics According to Amino Acid Patterns in the Core Region

**Figure 2:**
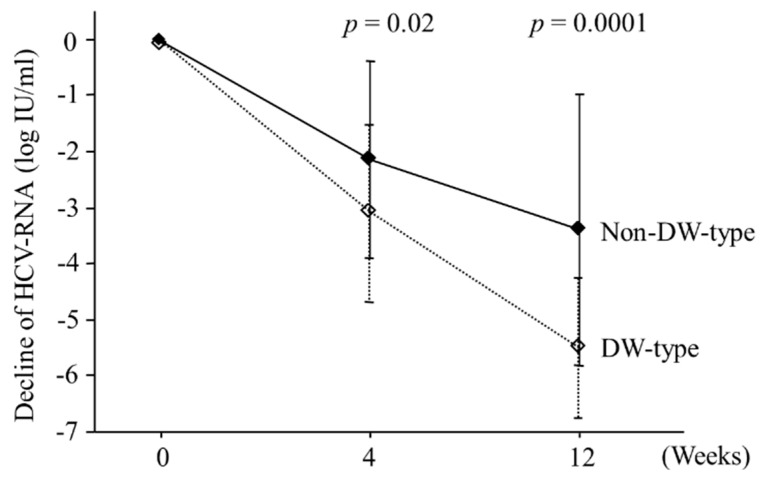
Viral kinetics according to amino acid mutations in the core region of hepatitis C genotype 1b patients treated with peginterferon plus ribavirin combination therapy. The mean decline in hepatitis C virus (HCV) RNA levels from baseline to weeks 4 and 12 during therapy are plotted. Vertical bars with terminal horizontal bars express standard deviation. Double wild (DW)-type: arginine and leucine at positions 70 and 91; non-DW-type: the other patterns of amino acids.

Figure 2 shows a decline in HCV RNA levels from baseline to weeks 4 and 12 during the combination therapy according to amino acid patterns at positions 70 and 91 in the core region. The fall in HCV RNA level at week 4 was significantly higher in patients with DW-type than in those with non-DW-type amino acid (−3.1 ± 1.6 *vs.* −2.1 ± 1.8 log IU/mL; *p* = 0.02): differences at week 12 were more clear (−5.5 ± 1.3 *vs.* −3.4 ± 2.4 log IU/mL; *p* = 0.0001).

### 2.4. Predictors of EVR as Determined by Univariate and Multivariate Analysis

Univariate analysis identified three parameters that correlated with EVR: platelet count (*p* = 0.03), amino acid patterns in the core region (positions 70 and 91, *p* = 0.002), and previous interferon treatment (*p* = 0.045). Multivariate analysis identified two parameters that independently influenced EVR: DW-type amino acid in the core region (*p* = 0.02) and platelet count ≥14 × 10^4^/mm^3^ (*p* = 0.02) (Table 1).

**Table 1:** Factors associated with early virologic response in this study identified by multivariate analysis.

Factor	Category	Odds Ratio (95% CI)	*p* Value
Platelet (/mm^3^)	≥14 × 10^4^	3.17 (1.17–8.60)	0.02
	<14 × 10^4^	1	
Core positions 70 and 91	DW-type	11.7 (1.46–94.5)	0.02
	Non–DW-type	1	

Double wild (DW)-type: arginine and leucine at positions 70 and 91, respectively, in the core region; non–DW-type: the other patterns of amino acids; CI: confidence interval.

### 2.5. Predictors of SVR as Determined by Univariate and Multivariate Analysis

Univariate analysis identified 8 parameters that correlated with SVR: age (*p* = 0.03), fibrosis stage (*p* = 0.01), hemoglobin level (*p* = 0.02), platelet count (*p* = 0.01), aspartate aminotransferase (AST)/alanine aminotransferase (ALT) ratio (*p* = 0.0001), core region amino acid patterns (positions 70 and 91, *p* = 0.004; position 110, *p* = 0.004), and number of ISDR mutations (*p* = 0.01). Multivariate analysis identified 6 parameters that independently influenced SVR: hemoglobin level ≥ 14 g/dL (*p* = 0.04), platelet count ≥ 14 × 10^4^/mm^3^ (*p* = 0.04), AST/ALT ratio < 0.9 (*p* = 0.009), DW-type amino acid at positions 70 and 91 (*p* = 0.02), non-wild-type amino acid at position 110 (*p* = 0.03), and 2 or more ISDR mutations (*p* = 0.02) (Table 2).

**Table 2:** Factors associated with sustained virologic response in this study identified by multivariate analysis.

Factor	Category	Odds Ratio (95% CI)	*p* Value
Hemoglobin (g/dL)	≥14	6.21 (1.07–35.9)	0.04
	<14	1	
Platelet (/mm^3^)	≥14 × 10^4^	5.15 (1.11–23.8)	0.04
	<14 × 10^4^	1	
Core positions 70 and 91	DW-type	6.76 (1.37–33.3)	0.02
	Non-DW-type	1	
Number of mutations in the ISDR	≥2	12.3 (1.41–107)	0.02
	<2	1	
Core position 110	Non-wild	14.5 (1.31–167)	0.03
	Wild	1	
AST/ALT	<0.9	6.17 (1.58–23.8)	0.009
	≥0.9	1	

Double wild (DW)-type: arginine and leucine at positions 70 and 91, respectively, in the core region; non-DW-type: the other patterns of amino acids; ISDR: interferon sensitivity determining region; CI: confidence interval.

### 2.6. Predictors of NR as Determined by Univariate and Multivariate Analysis

Univariate analysis identified two parameters that correlated with virological non-response: platelet count (*p* = 0.04) and non-DW-type at core region positions 70 and 91 (*p* = 0.005). Multivariate analysis confirmed these two parameters as independent factors (Table 3).

**Table 3:** Factors associated with virological non-response (NR) in this study identified by multivariate analysis.

Factor	Category	Odds Ratio (95% CI)	*p* Value
Platelet (/mm^3^)	≥14 × 10^4^	0.32 (0.12–0.86)	0.02
	<14 × 10^4^	1	
Core positions 70 and 91	DW-type	0.10 (0.012–0.79)	0.03
	Non-DW-type	1	

Double wild (DW)-type: arginine and leucine at positions 70 and 91, respectively, in the core region; non-DW-type: the other patterns of amino acids; CI: confidence interval.

### 2.7. Analysis of Factors Associated with DW-Type at Positions 70 and 91

**Figure 3:**
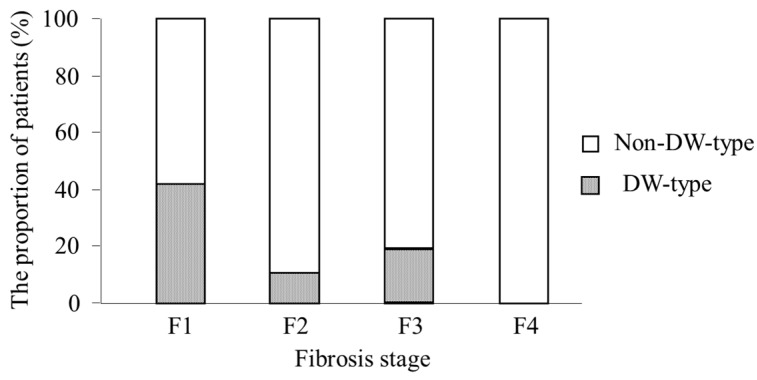
The proportion of patients with double wild (DW)-type or non-DW-type in the core region according to fibrosis staging. Patients showed a decreasing trend for the proportion of DW-type amino acid according to fibrosis stage (*p* = 0.01). DW-type: arginine and leucine at positions 70 and 91 in the core region; non-DW-type: the other patterns of amino acids.

We analyzed the relationship between the presence of the DW-type amino acid and patient profiles and found that fibrosis stage (*p* = 0.02) was the only factor that correlated with the presence of DW-type amino acid: patients showed a decreasing trend for the proportion of DW-type amino acid according to fibrosis stage (16/38 (42%) for F1, 3/27 (11%) for F2, 5/25 (20%) for F3, and 0/3 (0%) for F4; *p* = 0.01; Figure 3). The proportion of DW-type amino acid in patients with mild fibrosis (F1) was significantly higher than those with advanced fibrosis (≥F2) (16/38 (42%) *vs.* 8/55 (15%), *p* = 0.003).

## 3. Discussion

There are few studies on the relationship between the HCV core region and interferon-based treatment response because core amino acid is highly conserved and have been considered to be irrelevant in determining treatment response [9,21]. In line with this, most positions in the genotype 1b-core region were highly conserved except for 16 positions. In our study, amino acid mutation at positions 70, 91, and 110 was an independent and significant predictor of response to peginterferon plus ribavirin combination therapy. We also found that the DW-type, which has been negatively associated with development of hepatocellular carcinoma [22], was more frequent in patients with mild liver fibrosis.

It is unclear why these mutations are associated with response to interferon-based combination therapy. HCV core protein was shown to induce the expression of suppressor of cytokine signaling (SOCS) proteins known to inhibit interferon α action by blocking the Jak/STAT pathway [20]. Amino acid positions 70 and 91 are located in the amino-terminal hydrophilic portion of the core protein and in the regions for homotypic interaction (positions 36–91) [23]. The amino acid pattern at position 70 is independently associated with interferon therapy response [16,17]. This mutation has been also associated with response to triple therapy with protease inhibitor [24]. In an experimental study, amino acid mutation at position 91 was reported to play a role in enhancing internal initiation of HCV protein synthesis, leading to the expression of a core isoform, which may interact with viral and cellular components [25]. Tachi *et al.* reported substitutions of amino acid 70 was associated with the presence of steatosis which is common in chronic HCV infection and hepatic 8-hydroxy-2′-deoxyguanosine, a marker of oxidative stress, was higher in patients with methionine at amino acid 91 of the HCV core region [26]. Furthermore, we and several other groups have reported these mutations are associated with the risk of hepatocellular carcinoma [22,27,28]. These results suggest that examination of HCV core variation might be useful to help to understand the mechanism of interferon action as well as to predict treatment response and the risk of hepatocellular carcinoma in patients infected with HCV genotype 1b.

We found that the decline in HCV-RNA level was significantly more pronounced in patients with position 110 mutation (non-threonine) than in those without it (at 4 weeks, −3.66 ± 1.70 *vs.* −2.18 ± 1.69 log IU/mL, *p* = 0.01; at 12 weeks, −5.26 ± 1.53 *vs.* −3.71 ± 2.40 log IU/mL, *p* = 0.049, respectively). Moreover, we found that position 110 mutation was significantly associated with SVR in multivariate analysis (*p* = 0.03). Amino acid positions 101–121 comprise one of the highly basic regions [29], and residues 107–114 comprise one of the antigenic regions of the HCV core [30]. Additionally, a change from threonine to asparagine at position 110 was reported to decrease the reactivity of serum antibodies. Among other genotypes, variation at position 110 in genotype 2a was associated with treatment response [31]. Further studies are needed to determine the effect of this position on treatment response.

Previous studies suggested that the NS5A variation is correlated with response to interferon α therapy although some reported controversial results [13,14,17]. Enomoto *et al.* classified ISDR mutation into three groups according to number of mutations: wild-type (0 mutations), intermediate-type (1 to 3 mutations), and mutant-type (≥4 mutations) [13]. The present study showed that peginterferon plus ribavirin combination therapy achieved the SVR of 14/41 (34.1%) for wild-, 18/33 (54.5%) for intermediate-, and 6/8 (75%) for mutant-type groups. The mean number of ISDR mutations in patients with SVR was significantly higher than that in patients with non-SVR (1.5 ± 2.0 *vs.* 0.6 ± 1.0, respectively, *p* = 0.01). Furthermore, multivariate analysis revealed that the presence of ≥2 ISDR mutations was an independent factor associated with SVR to the combination therapy (Table 2).

The ISDR and an additional 26 carboxy-terminal amino acids of NS5A, the PKR-binding domain (PKRBD), have been shown to bind to and inactivate PKR *in vitro* [32,33]. Previous studies indicated that PKRBD and not the ISDR alone is a functional domain of NS5A and may confer resistance or sensitivity to interferon therapy [34,35,36]. We also examined the PKRBD and revealed that the number of mutations in the PKRBD, compared to the reference sequence of HCV-J [37], was significantly different between the SVR and non-SVR groups (6.02 ± 2.80 *vs.* 5.05 ± 1.44, *p* = 0.03).

With regard to host factors, our results showed that higher hemoglobin level, higher platelet count, and lower AST/ALT ratio were pretreatment predictors of SVR in line with previous studies [38,39]. Therefore, the combination of host factors and viral factors might be useful as pretreatment predictors of interferon-based treatment response to avoid unnecessary treatment for patients. Besides these factors, determination of IL28B genotype should also be useful to predict treatment response; however, genetic analysis would give rise to ethical issues and such data are not always available in clinical practice. IL28B genotype (rs8099917) was determined in 84 of 103 patients in the present study; TT and non-TT were 47 and 37, respectively [40]. This study was approved by the Ethics Committee of Chiba University (No. 406). The SVR rate in patients with IL28B TT was significantly higher than that with IL28B non-TT (51% *vs.* 16%, *p* < 0.001). Further study will be needed.

In summary, our results show that mutations at positions 70, 91, and 110 in the core region as well as ISDR/PKR-binding domain of the NS5A region could predict treatment response to peginterferon plus ribavirin in Japanese patients infected with genotype 1b-HCV. The variations in these regions might play important roles in interferon-based antiviral mechanism in HCV-infected patients.

## 4. Patients and Methods

### 4.1. Study Population

This retrospective study included 103 consecutive patients infected with HCV genotype 1b and high viral load (≥100 KIU/mL) treated with peginterferon α-2b plus ribavirin combination therapy between January 2005 and November 2007 at Chiba University Hospital in Chiba, Japan. Patients with chronic hepatitis B, autoimmune hepatitis, primary biliary cirrhosis, hemochromatosis, Wilson disease, or alcoholic liver disease were excluded from this study.

Patients received peginterferon α-2b at a median dose of 1.4 μg/kg (range, 0.7–2.2 μg/kg) subcutaneously each week plus oral ribavirin at a median dose of 11.4 mg/kg (range, 5.6–16.2 mg/kg) daily. Ribavirin dose was adjusted according to body weight (600 mg for ≤60 kg, 800 mg for >60 kg and ≤80 kg, and 1000 mg for >80 kg). Eighty-two of 103 patients received combined therapy for 48 weeks (standard treatment group), and the remaining 21 patients received combined therapy for 72 weeks or some other additional interferon treatments such as interferon beta or peginterferon α-2a monotherapy after the combined therapy (extended treatment group). Extended therapy was permitted upon patient request or in the case of slow responders defined as HCV RNA positive at 12 weeks of therapy. This study was approved by the Ethics Committee of Chiba University (No. 1462). Due to the retrospective nature of the study, informed consent was not obtained from all patients. Instead, participation in the study was posted at our institution.

Table 4 shows the clinical background of the patients at the start of the combination therapy.

**Table 4:** Clinicopathological features of patients infected with hepatitis C virus (HCV) genotype 1b at the start of peginterferon plus ribavirin combination therapy.

Characteristics	Number (%) or Mean ± SD
Number of Patients	103
Gender (male/female)	56/47
Age (years)	53.6 ± 11.4
Previous IFN treatment	38 (37%)
BMI (kg/m^2^)	23.3 ± 2.8
Fibrosis stage (F1/F2/F3/F4)	38/27/25/3
**Laboratory Data**	
AST (IU/L)	65 ± 61
ALT (IU/L)	79 ± 64
AST/ALT ratio	0.87 ± 0.25
Hemoglobin (g/dL)	14.5 ± 1.1
Leucocyte (/mm^3^)	5300 ± 1500
Platelet (× 10^4^/mm^3^)	16.2 ± 6.3
Total bilirubin (mg/dL)	0.9 ± 0.4
Serum creatinine (mg/dL)	0.7 ± 0.2
HCV RNA (log_10_ IU/mL)	6.3 ± 0.6
**Amino Acid Patterns in the Core Region**	
70 wild/70 non-wild	53/50
91 wild/91 non-wild	53/50
DW/Non-DW	26/77
110 wild/110 non-wild	14/89
**Number of Mutations in the ISDR**	
≥2/<2	21/82

Double wild (DW)-type: arginine and leucine at positions 70 and 91, respectively, in the core region; non-DW-type: the other patterns of amino acids; BMI, body mass index; IFN, interferon; AST, aspartate aminotransferase; ALT, alanine aminotransferase; ISDR, interferon sensitivity determining region; SD, standard deviation.

### 4.2. Definition of Treatment Response

Early virologic response (EVR) was defined as more than a 2-log decrease in HCV RNA during the first 12 weeks of therapy. SVR was defined as negative HCV RNA 6 months after treatment end. Virological non-response (NR) was defined as positive and less than a 2-log reduction of HCV RNA during treatment. EVR, SVR, and NR were analyzed on an intent-to-treat basis. Cases treated longer than 48 weeks were excluded from SVR analysis to reduce the impact of treatment protocol on therapeutic efficacy. EVR, SVR, and NR were evaluated in 99 (96%), 82 (80%), and 103 (100%) of the 103 patients, respectively.

### 4.3. Laboratory Tests

HCV RNA level was measured quantitatively before treatment, at weeks 4 and 12 during therapy, and 6 months after treatment end by Taqman PCR (Cobas Taqman HCV, Roche), and expressed using log_10_ IU/mL of viral loads, with the lower limit of 1.2 log_10_ IU/mL. HCV RNA genotype was determined by the method of Ohno *et al.* [41].

### 4.4. Histopathological Examination

Liver biopsy specimens were obtained percutaneously from 93 of 103 patients, and the specimens were histopathologically assessed according to the criteria of Desmet *et al.* [42]; fibrosis staging was defined as F1 (mild fibrosis), F2 (moderate fibrosis), F3 (severe fibrosis), and F4 (cirrhosis).

### 4.5. Nucleotide Sequences of the Core and NS5A Regions

HCV RNA was extracted from serum samples at treatment start and was reverse transcribed with random primers and SuperScript IΙΙ reverse transcriptase (Invitrogen, Carlsbad, CA, USA). Polymerase chain reaction (PCR) was performed using a HotStar Taq Master Mix kit (Qiagen, Hilden, Germany) and primers described previously to amplify the entire core region or the ISDR of the NS5A region [37,43]. PCR conditions were as follows: initial denaturation step at 95 °C for 15 min, followed by 45 cycles at 94 °C for 1 min, 45 °C for 1 min, and 72 °C for 3 min, and a subsequent extension for 7 min. The amplified PCR products were purified with a QIA quick PCR purification kit (Qiagen) after agarose gel electrophoresis. The purified DNA was sequenced using a Big Dye Terminator v3.1 Cycle Sequencing Kit (Applied Biosystems, Tokyo, Japan) and was determined with an ABI PRISM 310 Genetic Analyzer (Applied Biosystems).

Amino acid patterns in the core sequence were compared among the 103 patients. The most prevalent amino acid was defined as wild-type; wild-type at positions 70 (arginine) and 91 (leucine) was defined as double wild-type (DW-type) amino acid [15,40]. The number of amino acid mutations in the ISDR (position 2209 to 2248) was compared to the HCV-J sequence [37]. The sequences reported in this study will appear in the GenBank under accession numbers AB52390 to AB524005.

### 4.6. Statistical Analysis

Comparisons between groups were made by the χ^2^ or Fisher’s exact test for categorical variables and Student’s *t* test for quantitative variables. Quantitative data are expressed as mean and standard deviation. Univariate and multivariate logistic regression analyses were used to determine the predictors of EVR, SVR, and NR. Univariate analysis included the variables shown in Table 4. Variables with *p* values <0.05 on univariate analysis were entered into multivariate analysis to identify significant independent factors. The Cochran-Armitage trend test was used to analyze the association between the prevalence of amino acid mutations of the HCV core region and liver fibrosis of the subjects. Statistical significance was defined as *p* < 0.05.

## Abbreviations

HCV, Hepatitis C virus; ISDR, Interferon sensitivity determining region; DW-type, Double wild-type; SVR, Sustained virologic response; OR, Odds ratio; AST, Aspartate aminotransferase; ALT, Alanine aminotransferase; STAT, Signal transducer and activators of transcription; PKR, Double-stranded RNA-dependent protein kinase; EVR, Early virologic response; NR, Virological non-response; PCR, Polymerase chain reaction; SOCS, Suppressor of cytokine signaling; PKRBD, PKR-binding domain.
